# The Role of Serious Video Games in the Treatment of Disordered Eating Behaviors: Systematic Review

**DOI:** 10.2196/39527

**Published:** 2022-08-29

**Authors:** Wymann S W Tang, Tricia J Y Ng, Joseph Z A Wong, Cyrus S H Ho

**Affiliations:** 1 Yong Loo Lin School of Medicine, National University of Singapore Singapore Singapore; 2 Department of Psychological Medicine Yong Loo Lin School of Medicine National University of Singapore Singapore Singapore

**Keywords:** serious video games, serious games, video games, gamification, digital health, eHealth, mobile health, mHealth, disordered eating, eating disorders

## Abstract

**Background:**

Eating disorders and other forms of disordered eating cause significant complications and comorbidities in patients. However, full remission with current standard treatment remains low. Challenges to treatment include underdiagnosis and high dropout rates, as well as difficulties in addressing underlying emotion dysregulation, poor impulse control, and personality traits. Serious video games (SVGs), which have the advantages of being highly engaging and accessible, may be potential tools for delivering various forms of treatment in addressing the underlying psychopathology of disordered eating.

**Objective:**

This review aims to provide an overview of the possible mechanisms by which SVGs may affect the clinical course of disordered eating, while evaluating the outcomes of studies that have assessed the role of SVGs in the treatment of disordered eating.

**Methods:**

A systematic search was performed on PubMed, PsycINFO, and Embase, using keywords related to SVGs, disordered eating, and eating disorders. A narrative synthesis was subsequently carried out.

**Results:**

In total, 2151 papers were identified, of which 11 (0.51%) were included. Of these 11 studies, 10 (91%) were randomized controlled trials, and 1 (9%) was a quasi-experimental study. The types of SVG interventions varied across the studies and targeted different mechanisms of disordered eating, ranging from addressing problem-solving and emotion regulation skills to neurocognitive training for inhibitory control. Most (10/11, 91%) of the studies showed some benefit of the SVGs in improving certain physical, behavioral, or psychological outcomes related to disordered eating. Some (4/11, 36%) of the studies also showed encouraging evidence of the retention of these benefits at follow-up.

**Conclusions:**

The studies included in this review provide collective evidence to suggest the various roles SVGs can play in plugging potential gaps in conventional therapy. Nonetheless, challenges exist in designing these games to prevent potential pitfalls, such as excessive stress arising from the SVGs themselves or potential gaming addiction. Further studies will also be required to assess the long-term benefits of SVGs as well as explore their potential preventive, and not just curative, effects on disordered eating.

## Introduction

### Background

At least 9% of the world’s population is affected by eating disorders [1], with adolescents and young adults being the most likely to be diagnosed with eating disorders [2,3]. Besides their implications for mental health, eating disorders also cause multisystemic medical complications [4]. *Disordered eating* is a term that encompasses eating disorders that were formally defined by the Diagnostic and Statistical Manual of Mental Disorders, Fifth Edition, such as anorexia nervosa (AN), bulimia nervosa (BN), and binge eating disorder (BED) [5]. However, besides the aforementioned eating disorders, disordered eating may also refer to pathological eating behaviors, including restricting, bingeing, purging, or other compensatory behaviors, without fulfilling the Diagnostic and Statistical Manual of Mental Disorders, Fifth Edition, criteria for eating disorders [6]. Disordered eating attitudes can be driven by underlying body dissatisfaction or body image concerns (for restricting and purging), as well as an impairment of inhibitory control (for bingeing) [7,8].

Treatment of eating disorders hinges on early recognition and intervention, which is associated with better response to therapy as well as long-term outcomes [9]. According to guidelines from the National Institute for Health and Care Excellence, treatment of eating disorders such as AN, BN, and BED generally involves multidisciplinary effort, including psychoeducation, dietary rehabilitation, and monitoring and treatment of physical complications, as well as psychological treatment. Although eating disorder–focused cognitive behavioral therapy (CBT) is commonly perceived to be the first-line treatment and has the strongest and most rapid effects, evidence of its efficacy has been scarce for persistent AN as well as AN in adolescents [10]. Of note, a review published in 2002 revealed that only 46.8% of the patients with AN reached full recovery, 33.5% improved, and in 20.8% the disorder became chronic [11]. Treatment is also often limited by high rates of patients dropping out, ranging from 20.2% to 49.6% [12]. Mortality rates of these illnesses are still high, with those who received inpatient treatment for AN having more than 5 times increased mortality risk [13].

Challenges to the treatment of disordered eating are multifold. To start with, disordered eating is often underdiagnosed and undertreated because of a lack of awareness or feelings of shame associated with its diagnosis [14-16]. Obstacles to treating disordered eating can then be broadly considered in terms of patient- and clinician-related factors. Clinician-related factors that prevent use of evidence-based therapies include lack of training and individual beliefs regarding the effectiveness of certain forms of therapy, as well as over- or undervaluing certain elements of therapy [17]. There may also be difficulties in establishing a therapeutic alliance with the patient who is required to become vulnerable and give up some sense of control during the course of treatment [18]. Patient-related factors for poor treatment response can be examined through the underlying psychopathology of eating disorders. In patients with AN or BN, for instance, an underlying ego-syntonic pursuit of thinness or body dissatisfaction may be difficult for the patient to give up [18,19]. Furthermore, core features seen in disordered eating, such as the lack of impulse control, poor emotion regulation [20], and high reward-seeking behavior [21], are often difficult to address even with established forms of psychotherapy [22,23]. These comorbidities can also in turn affect motivation and compliance to psychological treatment [18]. In addition, traits of narcissistic, borderline, obsessive-compulsive, and avoidant personalities, which may be common in patients with disordered eating, may also negate treatment adherence and effectiveness [18,24]. The limitations of current conventional treatment as well as difficulties arising from the aforementioned factors necessitate a consideration of alternative treatment options or treatment options complementary to existing ones.

Technology is increasingly harnessed in the treatment of psychiatric conditions [25]. One example is the use of virtual reality (VR) to target clinical features of eating disorders such as binge eating, cravings, and body dissatisfaction through VR-mediated cue exposure and reference frame shifting [26]. According to a recent meta-analysis, VR-enhanced CBT has been shown to display better efficacy than CBT alone in reducing the frequency of binges and situation-induced body dissatisfaction [27]. VR has also been shown to be a feasible intervention to improve inhibitory control and thereby reduce binge eating episodes [28]. Internet-based CBT is another innovation that circumvents certain limitations of traditional CBT, such as high costs, long waiting time, and perceived stigma associated with seeking help for psychiatric disorders [29]. However, the challenges of high dropout rates and poor compliance remain [30].

This review is interested in the possible role of serious video games (SVGs) in addressing disordered eating. Games are by definition an activity in which “independent decision makers seek to achieve their objectives in a limiting context” [31]. Serious games can be simply viewed as games with a *serious* objective, often for education, vocational training, or problem-solving [32]. Nonetheless, the line between serious and entertainment games is sometimes blurred because certain entertainment games are sometimes repurposed for an educational purpose as well [33]. Serious games have been used in various fields, ranging from education and military applications to interpersonal communications training [32]. A key feature of these serious games is that besides delivering knowledge or skills to the player, they stimulate an environment through narrative story, gameplay, or encounters that is safe and controlled for users to be able to practice new learned skills or behaviors [34,35]. In today’s landscape, serious games are increasingly available on digitized platforms, ranging from mobile apps [36] to VR devices [35].

Serious games have had some early success in treating a range of psychiatric conditions and have been deemed to be highly feasible and acceptable to both patients and clinicians [34]. Psychiatric conditions in which serious games have been used to address symptoms include depression [37] and addiction problems such as substance abuse [38] and internet addiction [39]. It is noteworthy that these psychiatric conditions happen to be comorbidities commonly associated with disordered eating. One example of serious games being used in treating psychiatric conditions is Smart, Positive, Active, Realistic, X-Factor Thoughts (SPARX), a participative game based on CBT that targets depression in adolescents. The treatment outcomes of SPARX were shown to be comparable with those of conventional treatment despite its being a predominantly self-guided resource [40,41]. Dropout rates for this intervention were also low at approximately 9%, suggesting that such games may have an advantage in engaging patients belonging to the adolescent age group [41].

Serious games as a treatment can be highly engaging [42]. It has been shown that being appropriately challenged has positive effects on both engagement and learning, with the challenge of the game being a strong predictor of learning outcomes [43]. In serious games, players can advance through individualized game difficulty levels and be constantly challenged, allowing for personal growth [35]. By contrast, this may be difficult to replicate in traditional CBT. Furthermore, the potential of serious games in improving impulse control [44] and emotion regulation [45], which are core features in the psychopathology of disordered eating, would be one of their key foreseeable advantages. However, current studies examining the therapeutic effects of serious games on persons with disordered eating are sporadic. Hence, there is a need to systematically consolidate and critique such available studies to understand the effectiveness of serious games as a therapeutic medium for disordered eating behaviors.

### Objectives

This study aimed to answer the question regarding the effectiveness of SVGs in reducing disordered eating behaviors and addressing their underlying psychopathology. The review will provide an overview of the possible mechanisms by which SVGs can affect the clinical course of disordered eating, while evaluating the effectiveness of SVGs and their potential role in complementing current treatment options for disordered eating.

## Methods

### Search Strategy

The systematic review and meta-analysis were reported according to the PRISMA (Preferred Reporting Items for Systematic Reviews and Meta-Analyses) guidelines. Three databases (PubMed, Embase, and PsycINFO) were last searched on March 29, 2022, with no restrictions on publication dates. The following search terms were applied in the search strategy, with the use of relevant controlled vocabulary such as Medical Subject Headings, Emtree, and PsycINFO Thesaurus terms: *((serious gaming) OR (game) OR (computer-assisted therapy) OR (gamification) OR (gaming simulation) OR (video game) OR (applied game) OR (mobile game) OR (gamified application) OR (digital game)) AND ((eating disorder) OR (anorexia) OR (bulimia) OR (binge eating) OR (impulsive eating) OR (body image) OR (body dissatisfaction) OR (self-control) OR (inhibitory control))*.

### Selection of Articles

The inclusion criteria were as follows: studies (1) needed to be peer-reviewed randomized controlled trials (RCTs) or quasi-experimental design studies; (2) involved interventions delivered on a digital platform with gaming elements; (3) assessed the efficacy of SVGs in terms of their therapeutic benefit for eating disorders, disordered eating behaviors, and underlying body image concerns or inhibitory control; (4) involved populations prone to disordered eating; and (5) were in English.

Studies assessing the effects of gamified food-related inhibitory control training (ICT) were included because of evidence suggesting that interventions targeting food-related impulsivity have the potential to reduce binge eating frequency as well as address food cravings [46,47]. This is because disordered eating behaviors such as overeating have been linked to lack of inhibitory control, which is an underlying trait seen in eating disorders [8,48]. Studies examining the use of video games originally designed for entertainment purposes and not for their therapeutic effect were also included.

Studies were excluded if (1) the intervention was related to web-based CBT, web-based counseling, or guided self-help without elements of gamification; and (2) it involved VR-mediated interventions. Studies with VR-mediated interventions were excluded to isolate the effect of non–VR-related gamification on disordered eating as much as possible because of the extensive evidence of the efficacy of VR-mediated interventions [26-28].

The selection of articles was conducted by 3 authors (JZAW, TJYN, and WSWT). The selection was performed over 2 phases. In the first phase, articles were screened depending upon their relevance to this review based on their title and abstract. Shortlisted articles from the first phase of screening subsequently underwent full-text assessments for their eligibility to be included in this review. Disagreements on the selection process were resolved by discussion among the aforementioned authors as well as consultation with the senior author (CSHH).

### Review and Quality Assessment

The quality of each included study was independently evaluated by 2 separate authors (TJYN and WSWT) with any disagreements resolved through discussion. The RCTs were assessed using the Cochrane Risk-of-Bias 2 tool, whereas the noncontrolled experimental study was assessed using the Joanna Briggs Institute Critical Appraisal Checklist for Quasi-Experimental Studies [49,50].

### Data Extraction and Evaluation

After finalizing the selection of studies, the following information was extracted from the papers: (1) publication details (eg, title, authors, and country), (2) details of the SVG intervention (eg, type of platform, game objectives, and gameplay), (3) details of the studied population (eg, population type, sample size, and gender ratio), and (4) type of outcomes measured and the respective results. The studies were then categorized by the posited mechanisms by which the SVG intervention affected disordered eating.

The interventions used in the included studies were collectively assessed by adapting a framework developed by Murray et al [51] for evaluating the potential benefits of digital health interventions. The following factors were considered: (1) the accessibility of SVGs to populations with disordered eating, (2) causal explanations for how SVGs can treat disordered eating, (3) the key components required for SVGs to affect positive outcomes on disordered eating, (4) how target populations should be supported in the use of SVGs to treat or prevent disordered eating, (5) the possible harms of SVGs and the likelihood of their risks, (6) the costs of using SVGs incurred by users and the health system, and (7) the overall utility of SVGs. The evaluation of the SVGs was aided by a framework developed by Liverpool et al [52] that highlights key components contributing to engagement in digital mental health interventions among young people. This involves assessing the SVGs for intervention-specific factors, including suitability, usability, and acceptability, as well as user-specific factors, including motivation, opportunity, and capability [52].

## Results

### Search Results

**Figure 1:**
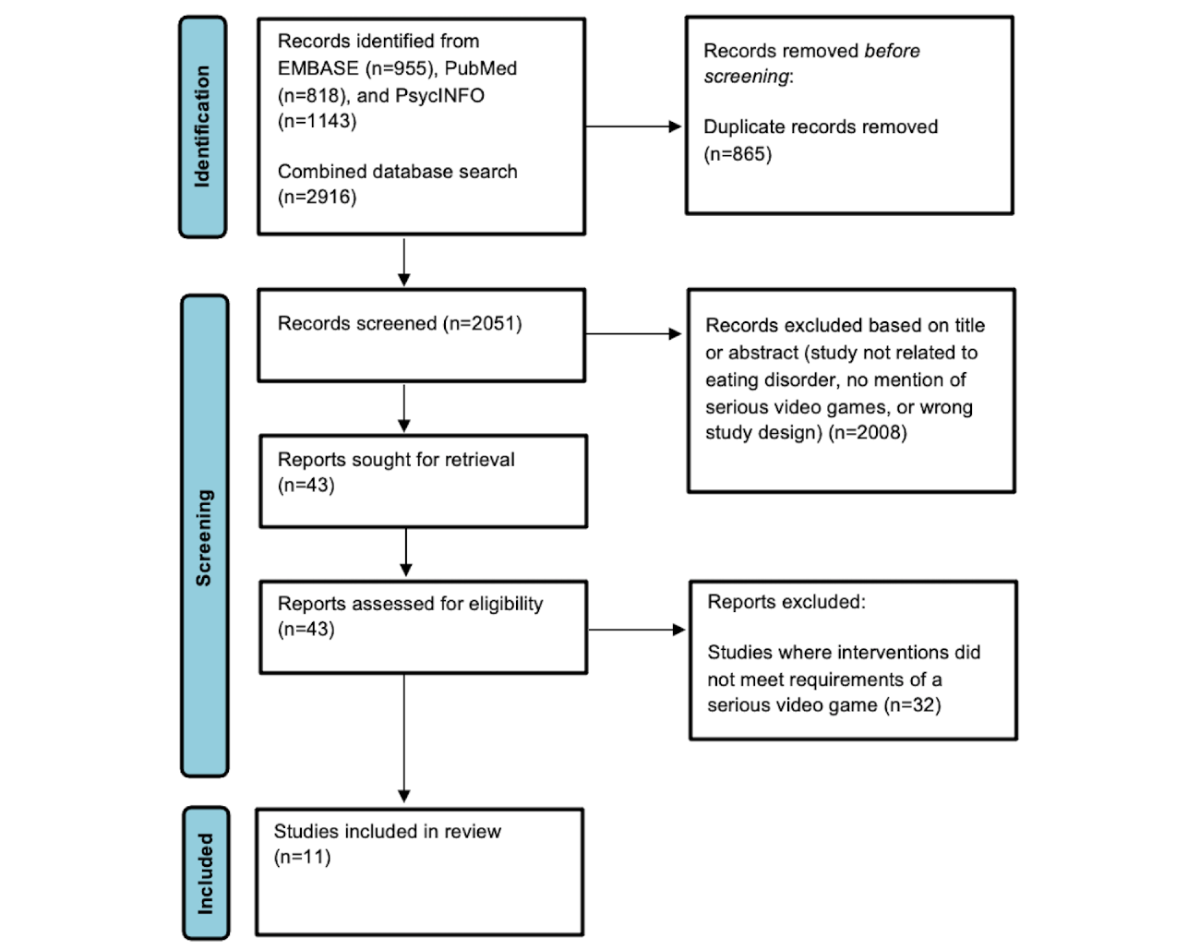
PRISMA (Preferred Reporting Items for Systematic Reviews and Meta-Analyses) flow diagram showing the selection of the studies.

Our search strategy yielded 2916 articles. Hand searching did not uncover other relevant studies. From the 2916 articles, 765 (26.23%) duplicates were removed. Of the remaining 2151 articles, we excluded 2108 (98%) after title and abstract screening because they did not include serious gaming as an intervention, or they focused on a disease unrelated to eating disorders. After full-text screening of the 43 remaining articles, 32 (74%) were excluded. Reasons for the exclusion of articles after full-text screening include not reporting outcomes related to eating disorders, assessing SVGs that were not run on digital platforms, and interventions not having gamified elements. Thus, of the initial 2916 articles identified, 11 (0.38%) were included in the systematic review. The PRISMA flowchart is presented in Figure 1. The main outcomes of the included articles are summarized in Table 1, whereas the details of the SVG intervention of each study are summarized in Table 2. The characteristics of the included studies are presented in Multimedia Appendix 1 [53-63], and the assessments of the risk of bias are presented in Multimedia Appendix 2 [54-63] and Multimedia Appendix 3 [53]. Of the 11 included studies, 10 (91%) were deemed to be at a low overall risk of bias, with the exception of 1 (9%) study, regarding which concerns were raised over the suitability of the outcome measures.

**Table 1:** Characteristics and outcomes of the included studies.

Serious video game category and title			Study, year	Outcome measures (physical, behavioral, and psychological)	Main outcomes
**Serious video games for emotion regulation skills**					
		The Use of Videogames as Complementary Therapeutic Tool for Cognitive Behavioral Therapy in Bulimia Nervosa Patients	Fernandez-Aranda et al [53], 2015	Frequency of bingeing and purging Dropout rates Eating disorder psychopathology Anxiety and anger Remission rate (partial or complete)	Intervention group achieved nonstatistically significant higher rates of total remission than the control group (50% vs 28%, respectively; *P*=.22) Intervention group had lower treatment attrition rates than the control group (20% vs 44%, respectively; Cohen d=0.54) and displayed improvements in emotion regulation, whereas the control group showed persisting emotion dysregulation
**Serious video games for body image concerns**					
	An App-Based Blended Intervention to Reduce Body Dissatisfaction: A Randomized Controlled Pilot Study		Kollei et al [54], 2017	Eating disorder psychopathology Depressive symptoms Body dissatisfaction	Intervention group showed significantly greater reduction in body dissatisfaction (Cohen d=–0.62; *P*=.001) and a medium-sized effect in reduction of eating disorder symptoms (Cohen d=–0.46; *P*=.007) which persisted at 1-month follow-up No significant effect on depressive symptoms was noted
	A Brief Mobile Evaluative Conditioning App to Reduce Body Dissatisfaction? A Pilot Study in University Women		Kosinski [55], 2019	Depressive symptoms Eating disorder psychopathology Self-esteem Body dissatisfaction and drive for thinness	No significant reduction of body dissatisfaction between the evaluative conditioning and control conditions was noted, but body dissatisfaction fell across conditions with a small effect size (*r*=0.27; *P*<.005). Similar patterns presented for the drive for thinness (*r*=0.67; *P*<.001) and self-esteem (*r*=0.29; *P*<.05) No statistically significant effects were observed for bulimia and restraint scores
	An Interactive Training Programme to Treat Body Image Disturbance		Gledhill et al [56], 2017	Eating disorder psychopathology Self-esteem Body size perception and body image concerns	Study 1 The intervention succeeded in shifting the thin-fat categorical boundary for individuals with body size concerns, as well as improved eating restraint (day 14 difference z score=0.92, 95% CI 0.33 to 1.51; *P*=.003), body weight (day 14 difference z score=1.15, 95% CI 0.58 to 1.72; *P*<.001), and body shape concerns (day 14 difference z score=1.04, 95% CI 0.45 to 1.64; *P*=.001) Study 2 The intervention succeeded in shifting the thin-fat categorical boundary significantly in participants with anorexia nervosa. Eating disorder symptoms also improved for at least a month (Eating Disorder Examination Questionnaire score day 1 vs day 30 difference z score=0.74, 95% CI 0.20 to 1.28; *P*=.008) The degree of body size category boundary shift was significantly correlated with changes in the eating disorder symptoms
	When You Exercise Your Avatar in a Virtual Game: The Role of Avatars’ Body Shape and Behavior in Users’ Health Behavior		Joo and Kim [57], 2017	Exercise and eating behavior	No significant effects of the avatars’ lifestyle on the participants’ exercising or eating behaviors were observed There was a significant positive effect of the normal-weight body shape of the avatars on the participants’ exercising behaviors (*P*=.02). No difference was noted for players who used the obese avatars
**Serious video games for neurocognitive training to influence eating behaviors**					
	App-Based Food-Specific Inhibitory Control Training as an Adjunct to Treatment as Usual in Binge-Type Eating Disorders: A Feasibility Trial		Keeler et al [58], 2022	Food valuation on palatability of high- and low-energy–dense foods Eating disorder psychopathology Depressive symptoms Anxiety Impulsivity	Intervention did not reduce binge eating frequency but showed greater reduction in eating disorder psychopathology (SES^a^=–0.57, 95% CI –1.12 to –0.03) and valuation of high-energy–dense foods than usual treatment (SES=–0.61, 95% CI –0.99 to –0.24). These effects were lost and reduced, respectively, at 8-week follow-up At 8 weeks, the intervention group showed greater reduction in food addiction symptoms and lack of perseverance with a small effect size (SES=–0.23, 95% CI –0.81 to 0.34)
	Gamified Working Memory Training in Overweight Individuals Reduces Food Intake but Not Body Weight		Dassen et al [59], 2018	BMI Food intake and healthy eating Self-control Dropout rate Executive function Eating disorder psychopathology	WM^b^ training did not result in significant additional weight loss WM training resulted in a significant reduction in caloric intake after training, especially at high levels of craving Both groups showed improvements in self-reported emotional eating and self-control
	Gender Differences in the Effect of Gamification on Weight Loss During a Daily, Neurocognitive Training Program		Forman et al [60], 2021	Weight Enjoyment of game and compliance to treatment Inhibitory control	Gamification had a significantly stronger effect on weight loss for men than for women No significant differences were observed between genders for the effect of gamification on enjoyment, compliance, and impulse control
	A Serious Game to Increase Healthy Food Consumption in Overweight or Obese Adults: Randomized Controlled Trial		Blackburne et al [61], 2016	Eating behavior Cognitive restraint Go–No-Go performance	Inhibitory control improved with the intervention, which was associated with increased consumption of healthy foods and reduced consumption of unhealthy foods. Cognitive restraint also improved
	Computerized Neurocognitive Training for Improving Dietary Health and Facilitating Weight Loss		Forman et al [62], 2019	Weight Frequency of food consumption Implicit preference for sweets	ICT^c^—both gamified and nongamified—were deemed acceptable and feasible Only participants with higher baseline implicit preference for sweets experienced weight loss benefits from ICT. However, gamification marginally reduced the impact of ICT
	Executive Function Training With Game Elements for Obese Children: A Novel Treatment to Enhance Self-regulatory Abilities for Weight-Control		Verbeken et al [63], 2013	BMI Treatment feasibility and acceptability Executive function Visuospatial WM Stop-signal task performance	The intervention showed significant effects in WM and meta-cognition and displayed significant improvements in weight loss maintenance at 8 weeks, although the effect was lost at 12 weeks No significant effects were observed for inhibition and the stop-signal task

^a^SES: standardized between-group effect sizes.

^b^WM: working memory.

^c^ICT: inhibitory control training.

**Table 2:** Characteristics of the serious video gaming interventions.

Game category and study					Game title		Serious game genre			Platform		Objectives		Gameplay
**Serious video games for emotion regulation skills**														
			Fernandez-Aranda et al [53], 2015		PlayMancer: Islands		Goal oriented and problem-solving	PC				To increase emotion–self-control skills and self-control over users’ general urgency to act		Players are immersed in the setting of an island and are required to overcome challenges by achieving therapeutic targets Biosensors and a camera that continuously tracks the emotional state of the player are used to monitor physiological changes in response to the players’ emotional state
**Serious video games for body image concerns**														
	Kollei et al [54], 2017			Mindtastic Body Dissatisfaction app		Cognition and brain training			Mobile app		Approach-avoidance training to foster approach of functional stimuli and avoidance dysfunctional stimuli		Players are shown (1) pictorial stimuli of their own bodies as well as that of their ideal bodies and (2) positive and negative body-related statements. They are required to pull the positive statements and pictures of themselves toward themselves and swipe away the negative statements and idealized pictures	
	Kosinski [55], 2019			Executive conditioning app		Cognition and brain training			Mobile app		Evaluative conditioning		The player’s photographs are taken to act as conditioned stimuli. Positive photographs that elicit a positive affective response and do not correspond to feminine ideals were used as unconditioned stimuli. Players are shown 3 conditioned stimuli and unconditioned stimuli pairings at the start and are required during the game to pick out their conditioned stimuli and unconditioned stimuli pair as quickly as possible	
	Gledhill et al [56], 2017			Perceptual training with two-alternative forced-choice decisions		Cognition and brain training			PC		Evaluative conditioning		Participants are presented with a series of computer-generated imagery images of women’s bodies and trained to judge the respective body size. Feedback was given to the participants on whether their responses were accurate. “Inflationary” feedback was given with the intent to shift their categorical boundary of a “fat” body shape by 2 body shape variations higher	
	Joo and Kim [57], 2017			The Sims 4		Goal oriented and problem-solving			PC		To increase emotion– self‐control skills and reduce general impulsive behaviors		A web-based life simulation game, The Sims 4, was used Players were assigned to either a normal weight or obese avatar. Players were then instructed to operate their avatars in a healthy (exercise and fresh foods) or unhealthy lifestyle setting (sedentary lifestyle and unhealthy foods)	
**Serious video games for neurocognitive training to influence eating behaviors**														
		Keeler et al [58], 2022		FoodT, an inhibitory control training app		Cognition and brain training			Mobile app		To increase inhibitory control		Players are presented with pictorial stimuli consisting of high-energy foods, low-energy foods, and filler items, accompanied by “Go,” or “No Go” cues. Participants are required to tap on the “Go” items and avoid tapping on the “No Go” items	
		Dassen et al [59], 2018		WM^a^ training		Cognition and brain training			Tablet computer or PC		Psychoeducation and WM training		Each session comprises 3 WM tasks in the setting of a restaurant involving visuospatial memory, backward digit span, and object memory	
		Forman et al [60], 2021		Go–No-Go training		Cognition and brain training			PC		To increase inhibitory control		Players are presented with pictorial stimuli consisting of healthy and unhealthy foods, accompanied by “Go,” and “No Go” cues, respectively. Participants are required to tap on the “Go” items and avoid tapping on the “No Go” items	
		Blackburne et al [61], 2016		“NoGo,” a Go–No-Go inhibitory control training app		Cognition and brain training			Mobile app		To increase inhibitory control		Players are shown stimuli of healthy and unhealthy foods. Each game consists of (1) Go–No-Go trials where the reaction timer starts counting down next to the image after it is shown and (2) stop trials where the timer counts down while the images change between categories	
		Forman et al [62], 2019		Go–No-Go training		Cognition and brain training			PC		To increase inhibitory control		The gamified inhibitory control training involved the task of moving in a grocery store as quickly as possible while choosing the correct foods. This required players to respond to frequently presented stimuli (healthy foods such as fruit and vegetables) and inhibit their responses to nonfrequent stimuli (high-sugar food)	
	Verbeken et al [63], 2013			“Braingame Brian,” an executive function training game		Cognition and brain training			PC		To increase executive function (inhibitory control and WM)		The game is set in a game world with a storyline where the character, Brian, is required to complete tasks involving (1) WM training where the player has to reproduce correctly a random sequence of rectangles lighting up and (2) inhibitory control training in the setting of a factory, including both go trials and stop trials	

^a^WM: working memory.

### Physical Outcomes

Of the 11 studies, 4 (36%) measured the effects of the gamified interventions on physical outcomes such as weight or BMI. All interventions were used in the context of treating overeating in individuals who were overweight [59,60,62,63]. Among the 4 studies, only 2 (50%)—the gamified ICT in the study by Forman et al [60] and the executive function (EF) training in the study by Verbeken et al [63]—reported statistically significant reduction in BMI after the serious game intervention. This effect, achieved in the study through an intervention centered on ICT was noted to be more significant in men than in women [60]. However, in another study by Forman et al [62], it was reported that gamification slightly reduced weight loss benefits compared with normal ICT [62].

Of note, the other studies in which a healthy increase in BMI would have been an ideal outcome in the context of undereating did not measure weight or BMI as a study outcome likely because of the short duration of their interventions.

### Behavioral Outcomes

In terms of behavioral outcomes, the studies reported a variety of outcomes such as frequency of binges and purges; food intake; enjoyment in using, and compliance to, the intervention; dropout rates; nonverbal communication; and drive for exercise. However, the reporting on the type of behavioral outcomes among the studies was not consistent.

Varying outcomes have been obtained in terms of eating habits and attitudes toward food. CBT coupled with a role-playing problem-solving SVG achieved neither a statistically significant increase in the reduction of bingeing or purging episodes nor an increase in the rates of total or partial clinical remission of BN [53]. In addition, an avatar-based SVG based on social cognitive theory was unable to influence the short-term eating behaviors of participants [57].

However, the study by Dassen et al [59] showed that gamified working memory (WM) training helped in reducing caloric intake, especially at high levels of craving [59]. In a different vein, the study by Blackburne et al [61] showed that a gamified ICT app improved inhibitory control that was associated with increased consumption of healthy foods and reduced consumption of unhealthy foods. Similarly, an app-based food-specific ICT in the study by Keeler et al [58] showed small-sized effects on a greater reduction in food addiction and medium-sized effects in participants’ valuation on the palatability of high-energy–dense food. However, the latter effect was diminished at later follow-up. The intervention also did not show greater reduction in binge eating frequency than usual treatment [58].

Dropout or attrition rates were measured in 27% (3/11) of the studies. The study by Fernandez-Aranda et al [53] showed that conventional CBT coupled with an SVG (*PlayMancer:* *Islands*) resulted in lower treatment attrition rates than CBT alone (20% vs 44%, respectively) [53]. In a different setting, the gamified WM training in the study by Dassen et al [59] demonstrated a participant dropout rate of 23% (21/91) across the interventional training and sham training groups using the same game. Gamified ICT in the study conducted by Forman et al [62] demonstrated an attrition rate of 14.8%, which did not differ significantly from the attrition rate in the nongamified ICT group.

### Psychological Outcomes

#### Overview

Across the studies included in the review, a variety of instruments were used to assess the features of eating disorders and their psychiatric comorbidities. Commonly applied questionnaires in the field of eating disorders, namely the Eating Disorder Inventory-2, Symptom Checklist–Revised, State-Trait Anxiety Index, and State-Trait Anger Expression Inventory-2, were used. In some (2/11, 18%) of the studies, throughout the duration of the treatment, patients kept a daily food and purging diary. Assessments were made before and after group therapy. The frequency of binges and purges was reported in the study by Fernandez-Aranda et al [53]; however, no statistically significant differences were found between the 2 clinical groups.

#### Eating Disorder Psychopathology

Of the 11 studies, 6 (55%) reported on participant scores on the eating disorder questionnaires [53-56,58,59]. Of these 6 studies, 4 (67%) displayed statistically significant improvements in eating disorder symptoms [54,56,58,59], although these effects were not always maintained at follow-up after a longer period [54,58].

#### Body Dissatisfaction and Body Size Perception

As it is an important psychopathological factor driving disordered eating, body dissatisfaction was reported on by 18% (2/11) of the studies, involving 113 participants in total. Both studies involved university students: Kollei et al [54] focused on students with significant body dissatisfaction, whereas Kosinski [55] focused on female students. Both interventions were based on approach-avoidance training aimed at fostering the avoidance of dysfunction stimuli and approach of functional stimuli. This was done by pairing participants’ photographs with positively conditioned stimuli. In both studies, participants in the intervention group reported significant reductions in body dissatisfaction, although the study by Kosinski [55] did not display a significant difference compared with the control group where neutral stimuli were used.

Although the study by Gledhill et al [56] did not measure body dissatisfaction directly as an outcome, it showed that a gamified perceptual training paradigm with inflationary feedback was successful in modifying perceptions on body size (measured by their thin-fat categorical boundary) in both individuals with body size concerns and patients with AN.

#### Gender Differences in Outcomes

Forman et al [60] assessed the associations between gender differences (between men and women) and the effectiveness of a gamified neurocognitive intervention program on weight loss and inhibitory control. Although gamification significantly enhanced weight loss in men more than in women, the association between overall effectiveness on inhibitory control and gender differences was unclear. The study also assessed participant enjoyment of the daily neurocognitive training program. Although there was no significant difference due to gender in the effect of enjoyment, women generally reported, with a small effect, higher enjoyment scores. By contrast, gamification did not seem to enhance men’s enjoyment of the intervention. The study also examined compliance to the treatment regimen with gamification. Although statistically nonsignificant, compliance to treatment regimen as a result of gamification improved in men but decreased in women [60].

## Discussion

### Principal Findings

The results of the review show an overview of the possible ways that SVGs or gamification of CBT interventions can effect change in the symptoms of disordered eating or its underlying psychopathology. These effects can be classified into physical, behavioral, and psychological outcomes. Among the papers that reported physical outcomes of BMI or weight change, it was found that the SVGs and gamified interventions potentially had some effect in improving weight. However, it may not be clear whether this effect can be truly attributed to the inclusion of gamification elements or whether it would still be retained in the nongamified intervention of the same type [62]. The duration of the interventions was also likely to be too short to identify the long-term effects of these SVGs on the participants’ weight. Behaviorally speaking, there were mixed results on the effect of the SVGs on desired changes in eating behavior. However, it is noted that gamified versions of neurocognitive training such as WM or ICT seemed to report more positive behavioral changes than the role-playing or avatar-based games. Psychologically speaking, it was observed that SVGs of different types such as those targeting body dissatisfaction, inhibitory control, or WM were all able to produce improvements in eating disorder symptoms or psychopathology [54,56,58,59]. This informs developers of future SVGs for disordered eating on potential game mechanisms and types.

### Key Components of SVGs and Their Mechanisms in Treating Disordered Eating Behaviors

The studies (n=11) included in this review comprised 11 different SVGs. As seen in Table 2, the characteristics of the SVGs examined in this review were varied and addressed different aspects of disordered eating with different mechanisms. Of the 11 SVGs, 2 (18%) were gamified adaptations of approach-avoidance and evaluative conditioning training based on the cognitive behavioral model of body satisfaction, aimed to foster approach of functional stimuli and avoidance of dysfunctional stimuli in body dissatisfaction [54,55]. In a similar vein, the SVG in the study by Gledhill et al [56] was also based on cognitive training and worked by calibrating participants’ perception of categorical definitions of body shape. Of the 11 SVGs, 4 (36%) aimed to improve inhibitory control regarding unhealthy foods through ICT with *Go–No-Go* (GNG) food stimuli [58,60-62], whereas in the study by Fernandez-Aranda et al [53], the SVG was an immersive role-playing game that required players to solve problems by using emotion regulation skills and achieving impulse control. Interestingly, Dassen et al [59] and Verbeken et al [63] recognized the role of WM and EF, respectively, in behavioral self-regulation and sought to assess the effect of gamified WM and EF training on food intake [59,63,64]. Finally, it is noted from the paper by Joo and Kim [57] that commercially available simulation games, such as *The* *Sims 4*, may potentially be helpful in influencing real-life health behaviors of users through their avatars’ body shape and health behaviors.

The paper by Fernandez-Aranda et al [53] was the only one that examined the effects of an SVG and CBT on eating disorders, specifically BN. The study examined the role the SVG was able to play in addressing the limitations of CBT in treating the underlying traits of eating disorders (namely emotion regulation and impulsivity) while harnessing its gamified elements to ensure compliance and increased accessibility. *PlayMancer:* *Islands* is an SVG specifically designed for this purpose. The gameplay is unique in that the facial expressions and physiological markers such as heart rate and respiratory rate were monitored as responses to the players’ emotional states. Undesirable emotional states would then correspond to increased difficulty in completing tasks. Through the game, participants are required to learn and demonstrate emotion–self-control skills. Although the intervention had limited effect on clinical remission of BN, it was successful in addressing eating disorder psychopathology and in reducing treatment dropout rates. This is corroborated by case studies and case series, which, although not included in this review, showed positive effects of *PlayMancer: Islands* on the intervention group participants’ impulse control, emotion regulation, anxiety, and novelty seeking, as well as various physiological measures, including a functional magnetic resonance imaging scan comparing engagement of brain areas, compared with those of healthy controls [65-67]. The case study by Giner-Bartolomé et al [66] even reported changes in eating behavior, such as the number of bingeing episodes. Although SVGs such as *PlayMancer: Islands* cannot be considered an alternative to replace conventional therapy, it is likely that they can be used effectively to supplement the limitations of CBT in addressing emotion regulation and personality traits underlying eating disorders [24]. Nonetheless, stronger evidence for this intervention may be required in studies with a larger sample size and longer duration of follow-up. It will also be worth assessing the effect of *PlayMancer: Islands* on eating disorders other than BN with common psychopathological features, such as AN or BED. The feasibility and accessibility of the SVG intervention would also be a future practical consideration, given the use of biosensors and facial recognition technology.

It is interesting to note that the majority (10/11, 91%) of the other SVGs reviewed differ from *PlayMancer: Islands* in that these other games seek to provide cognitive response training to modify and address the underlying perceptions that drive disordered eating as opposed to the goal-oriented and problem-solving nature of gameplay used in *PlayMancer: Islands*. Of note, body dissatisfaction, ICT, and WM were the main concepts targeted by the SVGs studied.

The studies by Kollei et al [54] and Kosinski [55] both showed significant effects of SVGs in improving symptoms of body dissatisfaction. The Mindtastic Body Dissatisfaction app in the study by Kollei et al [54] used approach-avoidance training where participants are conditioned to perform approach actions (swiping toward themselves) and avoidance actions (swiping away) in response to specific stimuli such as positive and negative body-related statements, respectively. This approach has previously been shown to modify biases toward food and alcohol and in reducing their consumption [68-70]. By contrast, the mobile app intervention in the study by Kosinski [55] used evaluative conditioning, a form of Pavlovian conditioning, where a change in behavior or response is induced when conditioned stimuli (participants’ own photographs) are paired to unconditioned stimuli (positive body image photographs). The evaluative conditioning approach is supported by the work of Aspen et al [71], whose study showed that pairing participants’ bodies with positive social stimuli and pairing other bodies with neutral stimuli resulted in improvements in body shape concerns and self-esteem, as well as reduced food restriction. Kollei et al [54] and Kosinski [55] collectively show the potential of gamified mobile app interventions in providing remote cognitive training to modulate adaptive body image attitudes, which can be seen to be highly feasible and accessible. As both studies examined the effects of the interventions on university students, it would be useful to understand the generalizability of these effects on other demographics, as well as persons formally diagnosed with eating disorders. Given the use of these mobile app interventions in university students not diagnosed with eating disorders, it may also be interesting to assess the potential role of SVGs in preventing eating disorders in populations considered to be at high risk who have not yet been formally diagnosed. Studies with a longer follow-up duration may be helpful to inform whether such games can reduce the incidence of eating disorder diagnoses.

Commonly compared with approach-avoidance training, GNG training is another form of motor response training aimed at changing behaviors. GNG training was the modality that was gamified in the *FoodT* app in the study by Keeler et al [58]; this app, when used by patients with BN and BED, showed a reduction in eating disorder pathology and diminished the perceived palatability of high-energy–dense foods. GNG training was also used in the intervention in the studies by Forman et al [60,62] and Blackburne et al [61]. Although used in different populations, the effect size on eating disorder pathology of the *FoodT* app was similar to that of the Mindtastic Body Dissatisfaction app in the study by Kollei et al [54]. Although the meta-analyses conducted suggested that GNG training may be more effective than approach-avoidance training in influencing food behaviors [72,73], it is difficult to compare the usefulness of both SVGs because they address different psychopathological drivers of disordered eating (poor inhibitory control and body dissatisfaction). The *FoodT* app also lends support to the feasibility of cognitive training for disordered eating through gamified mobile apps, and it may be interesting to see whether a combination of features from these cognitive training apps can lead to better outcomes.

### Accessibility and Feasibility of Using SVGs

In addition to the benefits SVGs may have in the treatment of disordered eating, they may have other advantages. Compared with conventional treatment, SVGs are likely to be more accessible to patients with disordered eating. First, there is a high degree of correspondence between the demographics of video game players and patients with disordered eating; 38% of the video game players in the United States fell within the age range of 18 to 34 years, whereas 20% were aged <18 years [74]. It is hence likely that SVGs will appeal more to patients with disordered eating. Furthermore, with more SVGs using mobile apps as a platform coupled with increasing smart device ownership, SVGs have the potential to reach more persons with disordered eating. Finally, SVGs have relatively low barriers to use and also offer users the option of being by themselves, which may help circumvent issues of trust with the therapist or personality-related comorbidities such as avoidance in patients with disordered eating [18,75]. Hence, these factors may address certain challenges in the treatment of disordered eating such as low treatment uptake rates or high dropout rates.

### Further Tailoring of SVGs and Supporting Their Use

The study by Forman et al [60] compares gender differences in the effect of gamification on weight loss. Eating disorders tend to affect women more than men; however, disordered eating can affect men as well. In general, most studies have samples that consist predominantly of women. The study provides a different perspective on gender differences in the effect of SVGs. Most video game users tend to be men [76], and men are reported to be more motivated by gaming elements than women, which is supported by Hassouneh and Brengman [77], whose study indicates that the effects of SVGs on women and men can differ. The study also found that compliance to SVG interventions tends to be higher in men than in women. In addition, it was reported that the gamification elements had more positive outcomes for men. This may inform future recommendations on the potential use of SVGs for the treatment of disordered eating based on gender, as well as expectations regarding their subsequent clinical effectiveness. However, the SVG assessed by Forman et al [60] used a gamified GNG training modality; hence, it is not clear whether the trend noted in this study would apply to other SVG genres. As discussed by Forman et al [60], it is plausible that the inclusion of other gaming elements such as more elaborate backstories, incentives, and components that require collaboration with other participants in these SVGs may contribute to their appeal to women [78-81]. Such features may serve to enhance user motivation as well as foster a sense of connection with peers facing similar struggles with disordered eating. This may further promote user engagement with the SVGs.

Although not explicitly addressed by the included studies, it is important to consider the concerns over privacy and confidentiality that users may have regarding such SVGs, especially in the context of treating disordered eating behaviors. A review by Borghouts et al [82] showed that concerns over the safety and privacy of disclosed information can act as a barrier to engagement in such digital mental health interventions [82]. On the flipside, assurance of anonymity in the use of the digital intervention encouraged engagement [52,82]. Hence, in the design and implementation of such SVGs, it would be necessary to make sure that appropriate safeguards are in place to protect sensitive user data. It has also been suggested that the use of trusted brand names and transparency as well as evidence of credibility would serve to increase engagement of young users with digital mental health interventions [52].

### Possible Risks and Adverse Outcomes

Despite the many benefits an incorporation of SVGs may confer to the treatment of disordered eating, it is important to note potential pitfalls where SVGs may complicate treatment. One such instance would be the situation where the inclusion of serious gamification elements such as player scores may inadvertently cause additional stress or cognitive load because of the participant’s drive to do well in the game and distract participants from the therapeutic intentions of the game [83,84]. This could also potentially cause further insult to the self-esteem of the participant or further diminish confidence and motivation in complying with treatment. This is supported by the study by Mekler et al [85], which suggests that gamification elements such as scores and leader boards may function more as an extrinsic source of motivation that increases performance quantity but do not have a significant relationship with competence or intrinsic motivation; for example, players may attempt the game multiple times for the simple object of passing a particular level while not necessarily improving in competency. This may be apparent in the study by Forman et al [62] where gamified ICT actually reduced the weight loss benefits derived from conventional ICT. This was posited by the authors to possibly be due to distraction from the core stimuli by gaming elements such as visuals, music, and sound effects, which may have reduced prepotent reward response, hence affecting inhibitory response.

Nonetheless, among the other SVGs assessed in this review, scoring systems and leader boards are not key elements and may not necessarily cause the aforementioned effect. Furthermore, games such as *PlayMancer: Islands*, which uses biosensors to track physiological and facial expression responses to emotional states, will be able to pick up states of distress in participants as well as obtain an objective measure of progress [53]. Hence, as more research is being conducted to assess the effects of gamification elements on motivation, careful design and implementation of gamification will be crucial.

Given the relatively short duration of the implementation of the SVGs studied in this review, it may be a concern that the effects of gamification such as the participants’ enjoyability, compliance, and improvements in psychopathology may be due in part to the novelty of the gamification experience [86,87]. This implies that the usefulness or effectiveness of an SVG can be lost over time as the novelty effect of gamification wears off. This may not be a significant problem if the gamified intervention is meant to be used only in the short term, but it will have to be taken into account if the serious game is expected to be part of a patient’s long-term treatment. As discussed by Hamari and Kovisto [88], the addition of social networking and community elements to the gamification process may be helpful in enhancing effectiveness as well as the participants’ willingness to use the service. These effects are positively related to positive recognition and reciprocity. Hence, it is plausible that implementation of SVGs in the setting of support groups for disordered eating could enhance and prolong their benefits, while promoting a sense of connectedness.

There may also be concerns over SVGs fueling other problems such as gaming addiction. This is because impulsivity traits such as urgency and lack of perseverance are both associated with eating disorders such as binge eating as well as with problems of addiction [89]. At the same time, problematic internet use has been shown to act as a predictor of eating disorders in a meta-analysis [90]. However, none of the SVGs (n=11) assessed in the review have reported addiction as an adverse effect. This is likely to be due to the short duration of the intervention, making addiction unlikely. However, this may have to be taken into consideration if the SVGs are implemented for a longer duration, and countermeasures such as additional supervision or imposing appropriate limits on the use of the games may be warranted.

### Overall Utility and User Engagement

Taking the aforementioned considerations into account, although most of the outcomes measured in the individual papers were different, they collectively provide evidence to suggest the various roles SVGs can play in plugging potential gaps left by conventional therapy. Fitting the aforementioned factors into the framework developed by Liverpool et al [52] to evaluate user engagement, SVGs fare well in intervention-specific factors such as suitability, usability, and acceptability. In terms of person-specific influencing factors, young users likely have ample capability to engage in the use of SVGs. Furthermore, the novelty of SVGs also contributes to good user motivation, although its sustainability requires further examination. Opportunities for the adoption of SVGs may also be limited by confidentiality concerns as well as a lack of a sense of connectedness with others.

### Strengths and Limitations

The strength of this study is that it provides a broad overview of the various ways in which gamification may be imbued in different types of interventions to address different psychopathological and clinical aspects of disordered eating; for instance, SVGs that address body dissatisfaction, which may be more prevalent in AN, and those that target inhibitory control, which may be associated more strongly with overeating, BED, or BN [7,48]. Furthermore, this study provides insight into the effectiveness of SVGs not only in eating disorders but also in disordered eating, which encompasses pathological eating behaviors beyond the strict diagnoses of eating disorders.

The limitations of this study are that only a small number of studies were included for analysis, providing limited amount of data and evidence for the assessment of the effectiveness of SVGs in disordered eating. Furthermore, the outcomes of the studies were varied, making it difficult to compare effect sizes among them. In addition, given the small sample sizes in the studies, there is a higher risk of bias and potential lack of representativeness. It was also observed that not all the included studies addressed populations who had received formal diagnoses of disordered eating but instead included participants from the general population or those who had other characteristics such as obesity, which may not be accurate proxies for disordered eating. Hence, it may be difficult to extrapolate the effects of the studied interventions to apply in populations with formal diagnoses of disordered eating behaviors. However, the studies in the review do show the promise that SVGs hold for effecting change in eating behaviors. Hence, it may be helpful for more studies to validate this effect on populations with disordered eating. It is also noted that the SVGs reviewed in this paper targeted a predominantly young population such as university students, with a large proportion of participants being women. As such, more evidence may be required to advance knowledge on the effectiveness of SVGs in disordered eating in other age groups as well as in men, given the knowledge that eating disorders in men also face significant underdiagnosis and undertreatment [15].

### Further Studies and Recommendations

We have identified some gaps in current research that could be addressed in subsequent studies. First, more RCTs need to be conducted to expand the evidence pool. The World Health Organization’s global strategy on digital health includes the recommendation that research is important in ensuring safety and accountability in the implementation of digital health interventions [91]. Hence, it is important to solidify our understanding of the efficacy of SVGs as a treatment intervention for disordered eating. Pending studies, such as the one pertaining to the Self-help, Integrated, and Gamified Mobile-Phone Application intervention for patients who were overweight with maladaptive eating behaviors, will add to our current understanding of gamified mobile apps for disordered eating [92]. Studies investigating the effectiveness of SVGs for disordered eating in different socioeconomic backgrounds, levels of computer literacy, genders, and ages may also be helpful.

As mentioned earlier, the longest study in this review was held only over 8 weeks. Hence, the long-term impact of the interventions and the retention of its beneficial effects might not be well understood and can be explored further. Learning about the long-term impact of SVGs in the context of disordered eating may then also provide clues to their potential role not only in treatment but also in prevention of disordered eating in persons with high-risk factors.

Some of the SVGs discussed were computer applications. However, in recent years, computer sales have been declining, and other devices such as smartphones are increasingly being used [93]. As such, it will be prudent for future studies to study games supported on such platforms, which promise greater accessibility.

### Conclusions

In conclusion, this review supports the effectiveness of SVGs in complementing the current standards of care in treating disordered eating. Although more research is needed to gain a better understanding of the long-term effects of SVGs on disordered eating and their effectiveness across different demographic groups, SVGs have shown that they are able to provide measurable short-term benefits by addressing the underlying psychopathological processes that drive behaviors of disordered eating. Nonetheless, challenges exist in developing effective SVGs for disordered eating such that while having to keep the games appropriately challenging and engaging, caution has to be taken in the game design to prevent potential adverse outcomes.

## Appendix

Multimedia Appendix 1 Characteristics of included studies.

Multimedia Appendix 2 Risk-of-bias assessment according to the revised Cochrane Risk-of-Bias 2 tool for randomized controlled trials.

Multimedia Appendix 3 Risk-of-bias assessment according to the Joanna Briggs Institute Critical Appraisal Checklist for Quasi-Experimental Studies.

## Abbreviations

AN: anorexia nervosa
BED: binge eating disorder
BN: bulimia nervosa
CBT: cognitive behavioral therapy
EF: executive function
GNG: Go–No-Go
ICT: inhibitory control training
PRISMA: Preferred Reporting Items for Systematic Reviews and Meta-Analyses
RCT: randomized controlled trial
SPARX: Smart, Positive, Active, Realistic, X-Factor Thoughts
SVG: serious video game
VR: virtual reality
WM: working memory
