# Supplementary material for: The Role of Serious Video Games in the Treatment of Disordered Eating Behaviors: Systematic Review
Source: J Med Internet Res. 2022 Aug 29;24(8):e39527. doi: 10.2196/39527 (PMC9468918; doi:10.2196/39527)
Supplement: Multimedia Appendix 1 [file jmir_v24i8e39527_app1.docx]

Supplemental table 1. Characteristics of included studies

| **Reference** | **Country** | **Study Type** | **Study Population** | **Sample Size** | **Intervention group size; Intervention** | **Control group size; control setting** |
| --- | --- | --- | --- | --- | --- | --- |
| **Serious games for emotional regulation skills** | | | | | | |
| Fernandez-Aranda et al (2015) | Spain | Quasi-experimental design study | Female patients diagnosed with BN | 38 | 20; CBT + SVG | 18; CBT only |
| **Serious games for body image concerns** | | | | | | |
| Kollei et al (2017) | Germany | RCT | University students with body dissatisfaction | 53 | 26; application to reduce body dissatisfaction | 27; waitlist control |
| Kosinski et al (2019) | France | RCT | Female university students | 60 | 30; Game-like evaluative conditioning (EC) application | 30; same game-like app with neutral stimulation |
| Gledhill et al (2017) | United Kingdom | Study 1: RCT  Study 2:  Case Series | Study 1: Female undergraduate students with significant body size concerns  Study 2:  Female patients diagnosed with  atypical AN | Study 1: 40  Study 2: 21 | Study 1: 20; Perceptual training paradigm with “inflationary” feedback  Study 2: 21, same as above | Study 1: 20; Perceptual training paradigm with feedback consistent to categorical boundaries at baseline  Study 2: NA |
| Joo et al (2017) | Korea | RCT | University students | 124 | 31; for each group in the game (normal weight avatar + healthy lifestyle, obese avatar + healthy lifestyle, normal weight avatar+ unhealthy lifestyle, obese avatar + unhealthy lifestyle) | |
| **Serious games for neurocognitive training to influence eating behaviours** | | | | | | |
| Keeler et al (2022) | United Kingdom | RCT | Patients with diagnoses of either BN or BED | 80 | 40; ICT app + Treatment as usual | 40; Treatment as usual |
| Dassen et al (2018) | Germany | RCT | Adults aged between 18 to 60 with BMI above 25 with motivation to achieve weight loss | 91 | 51; Working Memory (WM) training + psychoeducation about a healthy lifestyle | 40; Sham training with difficulty level kept constant at easy level + education |
| Forman et al (2020) | USA | RCT | Overweight individuals (BMI between 25-50 kg/m2 with high sugar consumption (>3 servings per day) | 76 | 36; Gamified ICT+ psychoeducation on healthy eating+ no-sugar-added dietary prescription | 40; non-gamified ICT + psychoeducation + no-sugar-added dietary prescription |
| Blackburne et al (2016) | Australia | RCT | Individuals who wanted to improve their eating habits | 52 | 26; ICT application for unhealthy eating, smoking and drinking | 26; Waitlist control |
| Forman et al (2019), | USA | RCT | Adults aged between 18 to 65 with BMI between 25 and 50 | 106 | 27; ICT Game  29; ICT Non-game  + psychoeducation on healthy eating  +  no-sugar-added dietary prescription | 27; Sham game  23; Sham Non-game  + psychoeducation on healthy eating  +  no-sugar-added dietary prescription |
| Verbeken et al (2013) | Netherlands | RCT | Overweight children between 9-14 years old | 44 | 22; Gamified executive function training (working memory and inhibition training + treatment as usual protocols | 22; Inpatient treatment as usual with CBT integrated |
