# Supplementary figures and images for: The Role of Serious Video Games in the Treatment of Disordered Eating Behaviors: Systematic Review
Source: J Med Internet Res. 2022 Aug 29;24(8):e39527. doi: 10.2196/39527 (PMC9468918; doi:10.2196/39527)

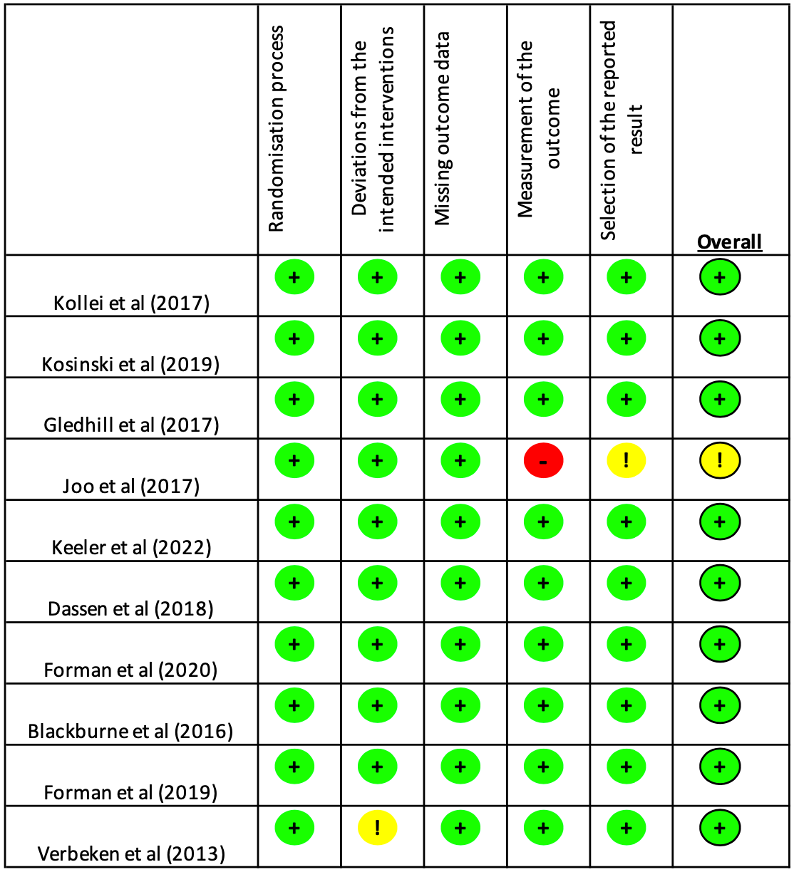

Supplement: Multimedia Appendix 2 [file jmir_v24i8e39527_app2.png]
