# Supplementary material for: The Role of Serious Video Games in the Treatment of Disordered Eating Behaviors: Systematic Review
Source: J Med Internet Res. 2022 Aug 29;24(8):e39527. doi: 10.2196/39527 (PMC9468918; doi:10.2196/39527)
Supplement: Multimedia Appendix 3 [file jmir_v24i8e39527_app3.docx]

Supplemental table 2. Risk of bias assessment according to JBI Critical Appraisal Checklist for Quasi-Experimental Studies

| Checklist question | Fernandez-Aranda et al (2015) |
| --- | --- |
| Is it clear in the study what is the ‘cause’ and what is the ‘effect’ (i.e. there is no confusion about which variable comes first)? | Yes |
| Were the participants included in any comparisons similar? | Yes |
| Were the participants included in any comparisons receiving similar treatment/care, other than the exposure or intervention of interest? | Yes |
| Was there a control group? | Yes |
| Were there multiple measurements of the outcome both pre and post the intervention/exposure? | Yes |
| Was follow up complete and if not, were differences between groups in terms of their follow up adequately described and analyzed? | Yes |
| Were the outcomes of participants included in any comparisons measured in the same way? | Yes |
| Were outcomes measured in a reliable way? | Yes |
| Was appropriate statistical analysis used? | Yes |
